# Elevated circulating TGFβ1 during acute liver failure activates TGFβR2 on cortical neurons and exacerbates neuroinflammation and hepatic encephalopathy in mice

**DOI:** 10.1186/s12974-019-1455-y

**Published:** 2019-04-02

**Authors:** Matthew McMillin, Stephanie Grant, Gabriel Frampton, Anca D. Petrescu, Elaina Williams, Brandi Jefferson, Alison Thomas, Ankita Brahmaroutu, Sharon DeMorrow

**Affiliations:** 1 Central Texas Veterans Health Care System, Temple, TX USA; 2 Department of Medical Physiology, College of Medicine, Texas A&M University Health Science Center, Temple, TX USA; 3 Division of Pharmacology and Toxicology, College of Pharmacy, The University of Texas at Austin, Austin, TX USA; 4 Department of Internal Medicine, Dell Medical School, The University of Texas at Austin, Austin, TX USA

**Keywords:** Acute liver failure, Azoxymethane, Microglia, Neuroinflammation, Necrosis

## Abstract

**Background:**

Acute liver failure resulting from drug-induced liver injury can lead to the development of neurological complications called hepatic encephalopathy (HE). Hepatic transforming growth factor beta 1 (TGFβ1) is upregulated due to liver failure in mice and inhibiting circulating TGFβ reduced HE progression. However, the specific contributions of TGFβ1 on brain cell populations and neuroinflammation during HE are not known. Therefore, the aim of this study was to characterize hepatic and brain TGFβ1 signaling during acute liver failure and its contribution to HE progression using a combination of pharmacological and genetic approaches.

**Methods:**

C57Bl/6 or neuron-specific transforming growth factor beta receptor 2 (TGFβR2) null mice (TGFβR2^ΔNeu^) were treated with azoxymethane (AOM) to induce acute liver failure and HE. The activity of circulating TGFβ1 was inhibited in C57Bl/6 mice via injection of a neutralizing antibody against TGFβ1 (anti-TGFβ1) prior to AOM injection. In all mouse treatment groups, liver damage, neuroinflammation, and neurological deficits were assessed. Inflammatory signaling between neurons and microglia were investigated in in vitro studies through the use of pharmacological inhibitors of TGFβ1 signaling in HT-22 and EOC-20 cells.

**Results:**

TGFβ1 was expressed and upregulated in the liver following AOM injection. Pharmacological inhibition of TGFβ1 after AOM injection attenuated neurological decline, microglia activation, and neuroinflammation with no significant changes in liver damage. TGFβR2^ΔNeu^ mice administered AOM showed no effect on liver pathology but significantly reduced neurological decline compared to control mice. Microglia activation and neuroinflammation were attenuated in mice with pharmacological inhibition of TGFβ1 or in TGFβR2^ΔNeu^ mice. TGFβ1 increased chemokine ligand 2 (CCL2) and decreased C-X3-C motif ligand 1 (CX3CL1) expression in HT-22 cells and reduced interleukin-1 beta (IL-1ß) expression, tumor necrosis factor alpha (TNFα) expression, and phagocytosis activity in EOC-20 cells.

**Conclusion:**

Increased circulating TGFβ1 following acute liver failure results in activation of neuronal TGFβR2 signaling, driving neuroinflammation and neurological decline during AOM-induced HE.

**Electronic supplementary material:**

The online version of this article (10.1186/s12974-019-1455-y) contains supplementary material, which is available to authorized users.

## Background

Drug-induced hepatotoxicity is a common cause of liver injury accounting for approximately one-half of the cases of acute liver failure [1]. The predominant extrahepatic complication of acute liver failure is the development of hepatic encephalopathy (HE), which can be defined as a brain dysfunction caused by liver insufficiency, manifesting as a wide spectrum of neurological or psychiatric abnormalities ranging from subclinical alterations to coma [2]. During acute liver failure, mortality is relatively high due to unpredictable systemic complications, with 20–25% of mortality resulting from increased intracranial pressure and the development of HE [3]. Therefore, strategies to manage symptoms of HE could prove beneficial in improving the overall survival rate of patients with acute liver failure. The increase in circulating and neural ammonia concentrations in HE promotes neurobehavioral changes like cerebral edema, astrocyte swelling, and dysregulation of numerous neurotransmitter systems [4]. Ammonia alone cannot explain all the neurobehavioral changes observed during HE. In animal models, arterial ammonia levels do not always correlate with the severity of the HE as neurological deficits can be observed prior to significant elevations of ammonia [5]. In addition, seizures can be associated with hyperammonemia but are not commonly observed in HE patients [6]. That being said, it is thought that hyperammonemia may be working in conjunction with neuroinflammation to cause the symptoms of HE [7, 8]. Indeed, it has been shown that microglia activation is observed in patients with acute liver failure [9]. We have demonstrated that activation of microglia occurs during HE in mice, and this is a result of increased chemokine ligand 2 (CCL2) and decreased chemokine (C-X3-C motif) ligand 1 (CX3CL1) expression in neurons, which has the combined effect of promoting microglia activation and neuroinflammation [10, 11]. Together, increased systemic inflammation and circulating inflammatory molecules observed during liver damage can contribute to neuroinflammation and the induction of encephalopathy.

Transforming growth factor beta (TGFβ) is a multifunctional family of cytokines consisting of 4 main proteins (TGFβ1–4). TGFβ binds to and activates a receptor complex comprising TGFβ receptor 1 and TGFβ receptor 2 (TGFβR2), to induce signal transduction pathways including SMAD-2/3. We have recently demonstrated that TGFβ1 is increased in the serum of mice with acute liver injury [12] and that this increased circulating TGFβ1 contributes to the pathological hyperpermeability of the blood–brain barrier that occurs in the development of HE [13]. Systemic treatment of mice with a pan-TGFβ neutralizing antibody had minimal effects on liver damage, but attenuated the development of HE [12], although the precise identity of the TGFβ family member responsible for these effects has not been defined. While we have demonstrated that there was increased TGFβ1 protein content and activation of downstream effectors of the TGFβ pathway in the brain after the development of HE, there was not a concomitant increase in TGFβ1 mRNA expression in the brain [12], suggesting that TGFβ1 is coming from an extracranial source.

The aims of this study were to determine if a more specific neutralizing antibody against TGFβ1 could attenuate neuroinflammation and the neurological complications of acute liver failure and to clearly delineate the role of TGFβ1 signaling in neuroinflammation and the development of HE using tissue-specific knockout mice and in vitro cell culture studies.

## Methods

### Azoxymethane model of acute liver failure

Floxed TGFβR2 mice (TGFβR2^fl/fl^, stock # 012603; Tgfbr2^tm1Karl^) from The Jackson Laboratory (Bar Harbor, ME) were crossed with Thy1-cre mice (stock # 007606; Tg(Thy1-cre/ERT2,-EYFP)AGfng) to generate a neuron TGFβR2 null mouse (TGFβR2^ΔNeu^). In vivo experiments were performed using male C57Bl/6 (TGFβR2^wt/wt^, 25–30 g; Charles River Laboratories, Wilmington, MA) or TGFβR2^ΔNeu^ mice. C57Bl/6 mice (TGFβR2^wt/wt^) were used as control mice after validation by assessing HE progression and time to reach coma in TGFβR2^wt/wt^, TGFβR2^fl/fl^, and TGFβR2^ΔNeu^ mice (see Additional file 1: Supplementary Materials and Methods). In addition, a methodology for genotyping and confirmation of TGFβR2 knockout in TGFβR2^ΔNeu^ mice is provided in Additional file 1: Supplementary Materials and Methods.

Acute liver failure and HE were induced via a single intraperitoneal injection of 100 mg/kg of azoxymethane (AOM) into mice as described previously [14–17]. In a sub-group of mice, neutralizing antibodies against TGFβ1 (anti-TGFβ1) (MAB240, R&D Systems, Minneapolis, MN) or immunoglobulin G1 (IgG1) (R&D Systems) were injected at 1 mg/kg into the peritoneum 1 h prior to AOM injection. Mice were injected with anti-TGFβ1 or IgG1 prior to AOM to ensure that TGFβ1 expression was not increased and no liver damage was present as a substantial liver injury is observed a few hours after AOM injection [5, 18]. For TGFβR2^ΔNeu^ mice, tamoxifen was injected into the peritoneum (0.125 mg/g/day for 3 days) prior to AOM injection with the last tamoxifen injection coming at 6 h after AOM injection. After AOM injection, mice were placed on heating pads set to 37 °C to ensure they remained normothermic. Hydrogel and rodent chow were placed on cage floors to ensure access to food and hydration. After 12 h and every 4 h thereafter, mice were injected subcutaneously with 5% dextrose in 250 μL saline to ensure euglycemia and hydration. Following injection, mice were monitored at least every 2 h (starting at 12 h post-AOM injection) for body temperature, weight, and neurological score using previously published methodology [10, 12, 19]. Once neurological impairment was evident, mice were continuously monitored with formal assessments of temperature, body weight, and neurological score performed each hour. The neurological score was assessed by an investigator blind to the treatments by assigning a score between 0 (absent) and 2 (intact) to each of the following parameters: the pinna reflex, corneal reflex, tail flexion, escape response, righting reflex, and ataxia. The summation of these six reflexes gives a neurological score between 0 and 12.

In a subset of AOM-treated mice, tissue was collected prior to neurological symptoms (pre-neurological), when minor ataxia and weakened reflexes were present (minor neurological) and when major ataxia and deficits in reflexes were evident (major neurological) as described previously [10, 20]. For all other mouse experiments, the endpoint was when mice lost their corneal and righting reflex, at which point the time to coma was recorded, the mice were euthanized, and tissue was collected.

### Cell culture

The HT-22 mouse hippocampal neuron cell line was provided by Dr. David Schubert (The Salk Institute, La Jolla, CA) and cultured using established protocols [21, 22]. HT-22 cells were treated with recombinant mouse TGFβ1 (0.5 ng/ml), GW788388 (1 μM), or SIS3 (10 μM). After 24 h, conditioned media was collected and cells were lysed for RNA isolation for subsequent RT-PCR assays.

A commercially available mouse microglia cell line (EOC-20) was purchased and cultured according to ATCC guidelines (Manassas, VA). Cells were plated onto 12-well plates and allowed to adhere overnight. After the cells were confluent, media was removed and replaced with conditioned media from HT-22 cells treated as outlined above. In addition, EOC-20 cells that were supplemented with HT-22 cell conditioned media were treated with C 021 dihydrochloride (C021) (1 μM), INCB 3284 dimesylate (INCB) (100 nM), or mouse soluble CX3CL1 (1 μg/ml). After 24 h, cells were lysed for RNA isolation and subsequent RT-PCR experiments.

### Phagocytosis assay

In EOC-20 cells, a Vybrant™ Phagocytosis Assay Kit (Molecular Probes, Eugene, OR) was used according to the manufacturer’s protocols. EOC-20 cells were plated into black 96-well cell culture plates at 10,000 cells per well and allowed to adhere overnight. Cell culture media was then removed and replaced with 100 μl conditioned media from HT-22 cells and subsequently treated with C021, INCB, or mouse soluble CX3CL1 for 16 h. Fluorescent *E. coli* bioparticles were resuspended in HBSS to a concentration of 1 mg/ml, and 50 μl was added to each well for 2 h at 37 °C. The conditioned media and fluorescent *E. coli* bioparticles were aspirated and fluorescence of *E. coli* bioparticles that did not undergo phagocytosis was quenched by adding 50 μl of trypan blue (0.125 mg/ml) per well. Trypan blue was aspirated, and fluorescence of internalized *E. coli* bioparticles was read at 480 nm excitation and 520 nm emission.

### Immunofluorescence

Frozen liver sections were cut into 8-μm sections and mounted onto positively charged slides. Liver sections were blocked with 5% goat serum and incubated with antibodies against TGFβ1 (Abcam, Cambridge, MA) or CK8 (Santa Cruz Biotechnology, Dallas, TX). Fluorescent secondary antibodies labeled with Dylight 488 and Cy3 (Jackson ImmunoResearch Laboratories Inc., West Grove, PA) were used, and the slides were counterstained with ProLong© Gold Antifade Reagent containing 4′,6-diamidino-2-phenylindole (DAPI) (ThermoFisher Scientific, Waltham, MA).

Free-floating immunofluorescence staining was performed on 30-μm brain sections. Brain sections were blocked with 5% goat serum and were subsequently incubated with antibodies against IBA1 (Wako Chemicals USA, Richmond, VA) to detect morphology and relative staining of microglia. Immunoreactivity was visualized using fluorescent secondary antibodies labeled with Cy3. Brain sections were then subsequently moved to positively charged slides and had coverslips mounted using ProLong© Gold Antifade Reagent containing DAPI.

Liver and brain sections were viewed and imaged using a Leica TCS SP5-X inverted confocal microscope (Leica Microsystems, Buffalo Grove, IL). Field fluorescence area of IBA-1 was determined by converting images to grayscale, inverting their color and quantifying field staining intensity with ImageJ software.

### Gene expression analyses

RNA was extracted from cell culture lysates or liver tissue using a RNeasy Mini Kit (Qiagen, Germantown, MD) according to the manufacturer’s instructions. Synthesis of cDNA was accomplished using a Bio-Rad iScript™ cDNA Synthesis Kit (Hercules, CA). RT-PCR was performed as previously described [23] using commercially available primers designed against mouse TGFβ1, CCL2, CX3CL1, interleukin-1 beta (IL-1β), tumor necrosis factor alpha (TNFα), and glyceraldehyde 3-phosphate dehydrogenase (GAPDH) (SABiosciences, Frederick, MD). A ΔΔCT analysis was performed using vehicle-treated tissue or untreated primary neurons as controls for subsequent experiments [24, 25].

### Protein expression analyses

Liver or cortex tissue was homogenized using a Miltenyi Biotec gentleMACS™ Dissociator (San Diego, CA), and total protein was quantified using a ThermoFisher Pierce™ BCA Protein Assay kit (Waltham, MA). The ThermoFisher TGFβ1 ELISA kit was performed in tissue or serum according to manufacturer’s instructions except the acid activation step was skipped to measure only bioactive TGFβ1 in our samples. For the Mouse Duoset CCL2, CX3CL1, IL-1β, and TNFα ELISA kits (R&D Systems), capture antibodies were incubated overnight in 96-well plates. After incubation, the assay was performed according to the instructions provided by R&D Systems. For each ELISA kit, the total input for each sample was 100 μg of protein or 100 μL of undiluted conditioned cell media. Absorbance was read using a SpectraMax® M5 plate reader from Molecular Devices (Sunnyvale, CA). Data were reported as TGFβ1, CCL2, CX3CL1, IL-1β, or TNFα concentration per milliliter of serum, per milligram of total lysate protein or per milliliter of conditioned cell media.

### Liver histology and serum chemistry

Paraffin-embedded livers were cut into 4-μm sections and mounted onto positively charged slides (VWR, Radnor, PA). Slides were deparaffinized and stained with Hematoxylin QS (Vector Laboratories, Burlingame, CA) followed by staining with eosin Y (Amresco, Solon, OH) and rinsed in 95% ethanol. The slides were then dipped into 100% ethanol and subsequently through two xylene washes. Coverslips were mounted onto the slides using CytoSeal XYL mounting media (ThermoFisher). The slides were viewed and imaged using an Olympus BX40 microscope with an Olympus DP25 imaging system (Olympus, Center Valley, PA). All images are taken as × 200 magnification.

Liver function was assessed by measuring serum alanine aminotransferase (ALT) and aspartate aminotransferase (AST) levels using a Catalyst One serum chemistry analyzer from IDEXX Laboratories, Inc. (Westbrook, MA).

### Statistical analyses

All statistical analyses were performed using GraphPad Prism software (GraphPad Software, La Jolla, CA). Results were expressed as mean ± SEM. For data that passed normality tests, significance was established using the Student *t* test when differences between two groups were analyzed, and analysis of variance when differences between three or more groups were compared followed by an appropriate post hoc test. If tests for normality failed, two groups were compared with a Mann-Whitney *U* test or a Kruskal-Wallis ranked analysis when more than two groups were analyzed. For the neurological score analyses, a two-way analysis of variance was performed followed by a Bonferroni multiple comparison post hoc test. Differences were considered significant for *p* values less than 0.05.

## Results

### Hepatic TGFβ1 is increased during AOM-induced HE

**Fig. 1:**
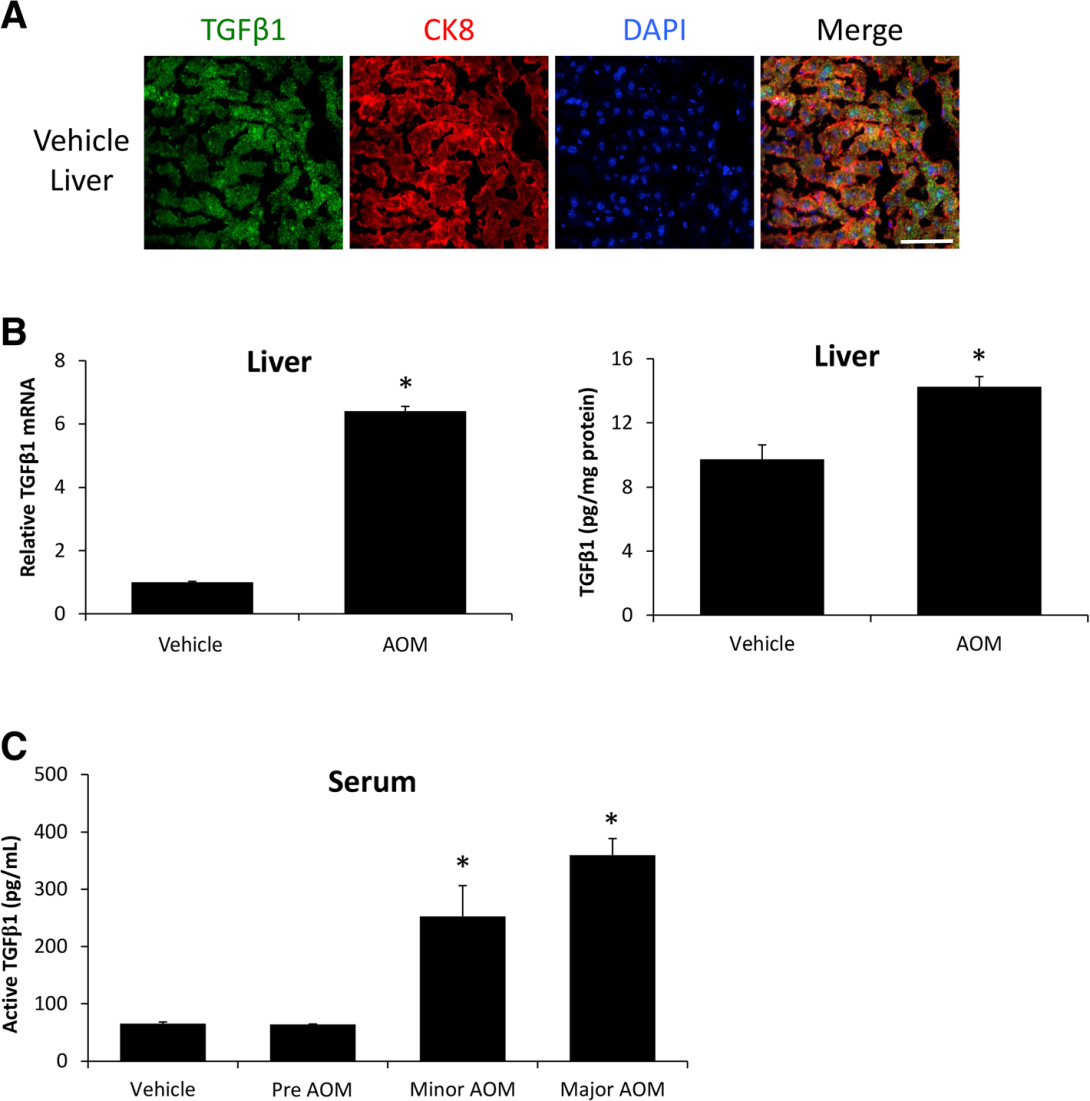
Hepatic TGFβ1 is increased during AOM-induced HE. **a** Immunofluorescence for TGFβ1 (green) and CK8 (red) in mice injected with saline. Scale bar indicates 50 μM. **b** Relative TGFβ1 mRNA expression in the livers from vehicle- and AOM-treated mice. **c** Concentration of active TGFβ1 levels in liver homogenates normalized by total protein concentration in vehicle and AOM-treated mice. **d** Serum active TGFβ1 concentration measured in picograms per milliliter of serum from vehicle and AOM-treated time course mice. *p < 0.05 compared to vehicle-treated mice. *n* = 3 for TGFβ1 mRNA analyses and *n* = 4 for ELISA analyses

TGFβ1 cellular localization in the liver was determined using immunofluorescence, demonstrating that TGFβ1 was expressed in vehicle-treated liver and that its expression co-localized with the hepatocyte marker CK8 (Fig. 1a). In order to validate that TGFβ1 is increased during AOM-induced HE, TGFβ1 expression was assessed in the livers of vehicle- and AOM-treated mice. Hepatic TGFβ1 mRNA expression was significantly increased in AOM-treated mice compared to vehicle-treated mice (Fig. 1b). This result translated to a significant increase of active TGFβ1 levels in the liver of AOM-treated mice compared to vehicle-treated mice (Fig. 1c). Circulating levels of active TGFβ1 were significantly increased in AOM-treated mice at the minor and major stages of neurological decline in comparison to vehicle-treated mice (Fig. 1d).

#### Neutralizing circulating TGFβ1 attenuates neurological decline and inflammation

**Fig. 2:**
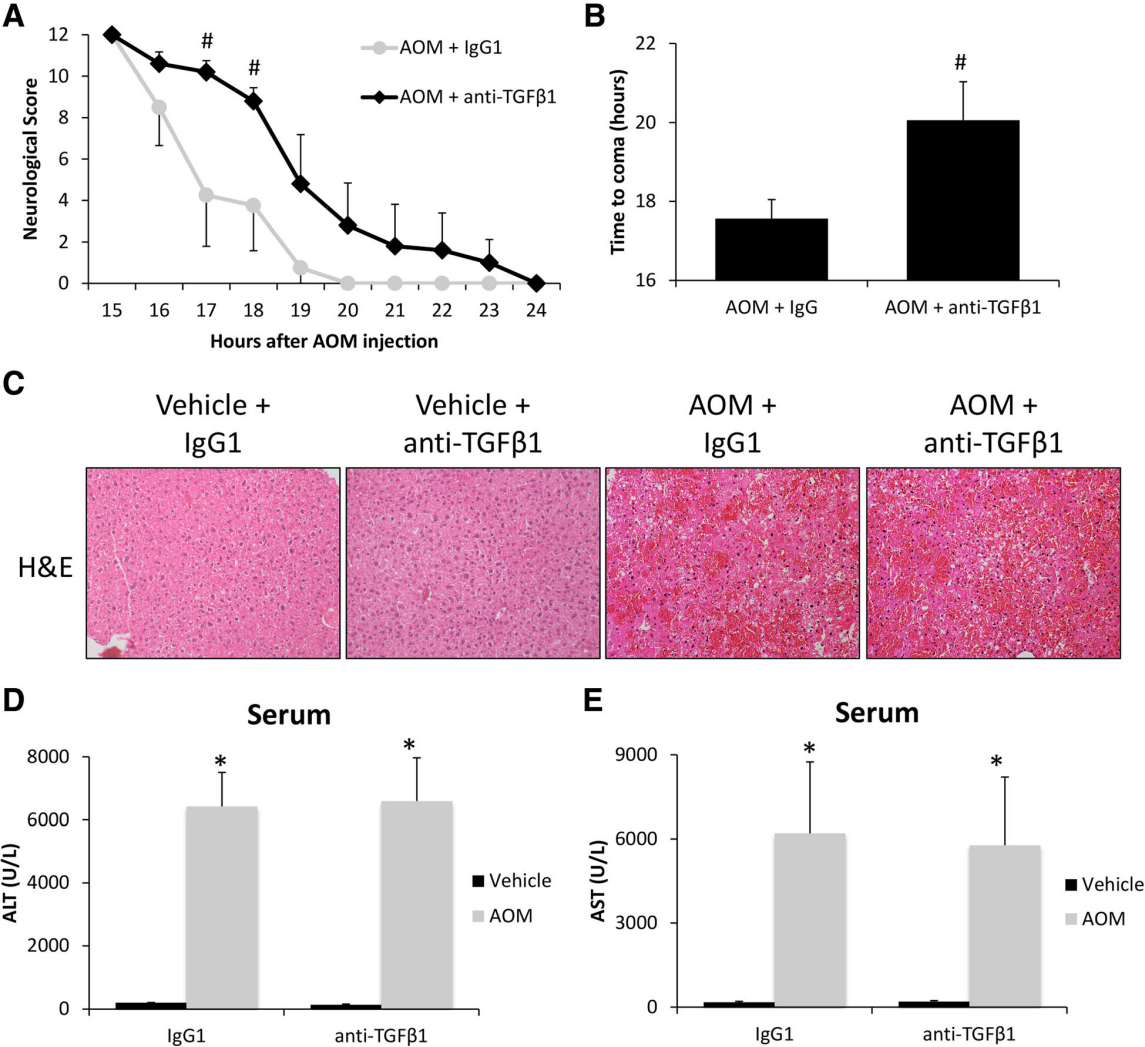
TGFβ1 contributes to HE-induced neurological decline. **a** Neurological score analyses as assessed by reflex scores and ataxia at the indicated hours post-AOM injection in AOM-treated mice pre-treated with IgG1 or anti-TGFβ1. **b** Time taken to progress to coma in hours of AOM-treated mice infused with IgG1 or anti-TGFβ1. **c** Representative H&E images from the livers of vehicle- or AOM-treated mice pre-treated with IgG1 or anti-TGFβ1. **d** Serum ALT levels of vehicle- or AOM-treated mice pre-treated with IgG1 or anti-TGFβ1. **e** Serum AST levels of vehicle- or AOM-treated mice pre-treated with IgG1 or anti-TGFβ1. *p < 0.05 compared to vehicle and IgG1-treated mice, ^#^*p* < 0.05 compared to AOM- and IgG1-treated mice. For all analyses, *n* = 4 for AOM-treated with IgG1 groups and *n* = 5 for AOM and anti-TGFβ1 groups.

In order to determine the specific effects of TGFβ1 on the progression of AOM-induced HE, a neutralizing antibody against TGFβ1 (anti-TGFβ1) was injected into mice 1 h prior to vehicle or AOM injection. Pre-treatment with anti-TGFβ1 was found to significantly reduce the rate of neurological decline in AOM-treated mice compared to AOM-treated IgG1 controls (*p* = 0.0268) with significant differences between the two groups at 17 h and 18 h after AOM injection (Fig. 2a). Latency to reach coma in AOM-treated mice injected with anti-TGFβ1 was significantly greater compared to IgG1-injected AOM-treated mice (Fig. 2b). This treatment had little effect on liver injury with significant hepatocyte necrosis and microvesicular steatosis observed in AOM-treated mice pretreated with anti-TGFβ1 or IgG1, and no apparent hepatic injury was observed in either vehicle group (Fig. 2c). In support of this finding, AOM treatment significantly increased the serum transaminases ALT (Fig. 2d) and AST (Fig. 2e) levels with no significant protection conferred by anti-TGFβ1> pre-treatment.

**Fig. 3:**
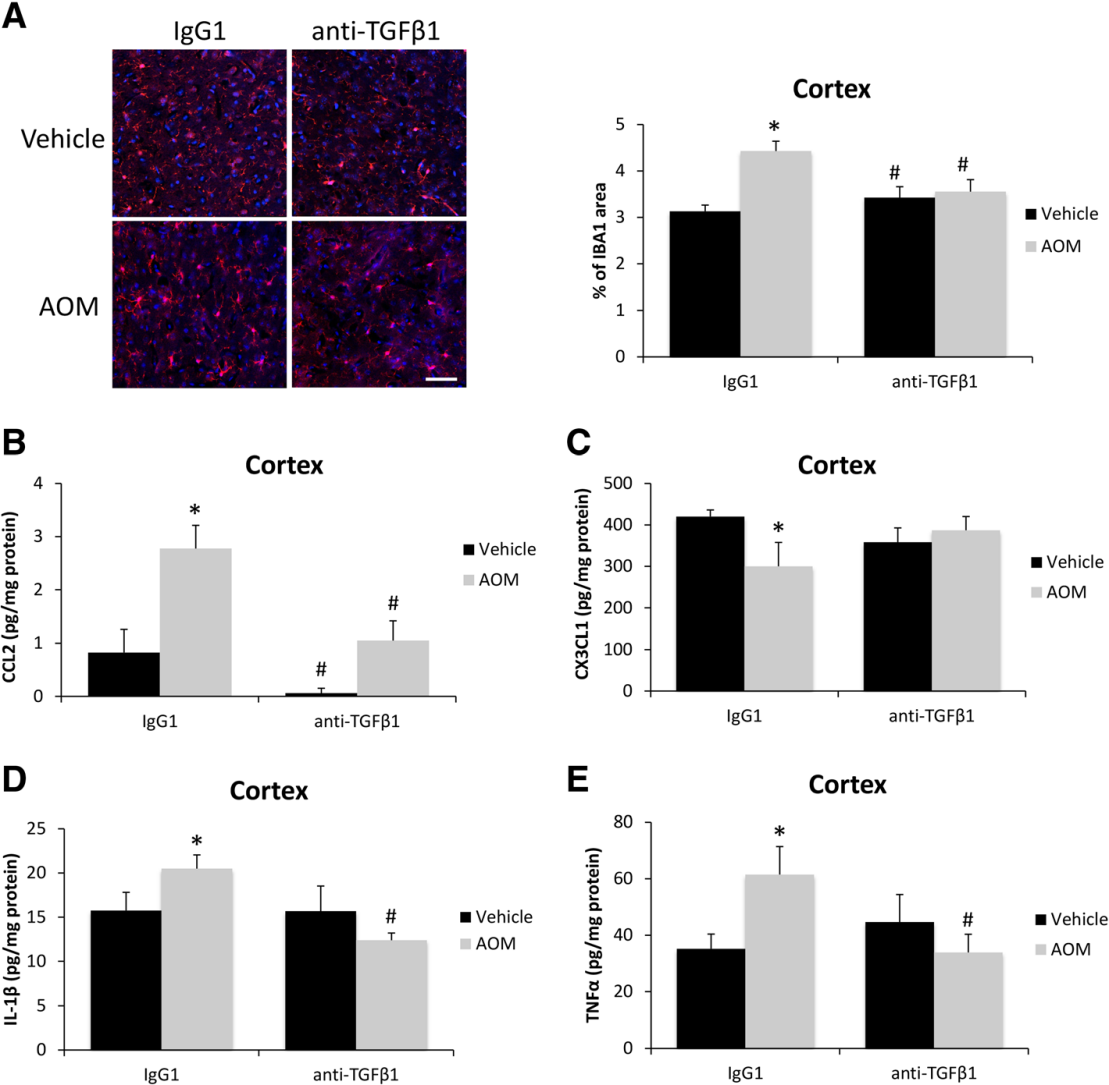
TGFβ1 promotes microglia proliferation and neuroinflammation. **a** Representative staining and quantification for IBA1 (red) in the cortex from IgG1 or anti-TGFβ1 mice administered saline (vehicle) or AOM. DAPI (blue) was used to stain nuclei. Scale bar indicates 50 μM. **b** CCL2 protein expression normalized by total protein concentration in the cortex of IgG1 or anti-TGFβ1 mice administered vehicle or AOM. **c** CX3CL1 protein expression normalized by total protein concentration in the cortex of IgG1 or anti-TGFβ1 mice administered vehicle or AOM. **d** IL-1β protein expression normalized by total protein concentration in the cortex of IgG1 or anti-TGFβ1 mice administered vehicle or AOM. **e** TNFα protein expression normalized by total protein concentration in the cortex of IgG1 or anti-TGFβ1 mice administered vehicle or AOM. *p < 0.05 compared to vehicle- and IgG1-treated mice, ^#^*p* < 0.05 compared to AOM- and IgG1-treated mice. *n* = 3 for all analyses

As this treatment had little effect on the liver but reduced the rate of neurological decline, it is logical to conclude that anti-TGFβ1 treatment is primarily influencing the neurological aspects of HE. As previously described, inflammation is a significant contributor to HE progression, and therefore, we assessed neuroinflammation in these animal models. Microglia proliferation, as assessed by IBA1 staining, was significantly increased in the AOM-treated mice pre-treated with IgG1 but not in the AOM-treated mice pre-treated with anti-TGFβ1 (Fig. 3a). There was a significant increase of CCL2 protein in the cortex of AOM-treated mice with a significant reduction in the mice treated with anti-TGFβ1 (Fig. 3b). In addition, CX3CL1 was significantly reduced in the cortex of AOM-treated mice treated with IgG1 but not in AOM-treated mice injected with anti-TGFβ1 (Fig. 3c). The pro-inflammatory cytokines IL-1β (Fig. 3d) and TNFα (Fig. 3e) were significantly increased in the cortex of AOM-treated mice pre-treated with IgG1 but not in AOM-treated mice pre-treated with anti-TGFβ1. These data support that antagonizing circulating TGFβ1 reduces microglia proliferation and the subsequent pro-inflammatory response resulting in a reduction of neurological decline.

#### Neuronal TGFβR2 promotes neuroinflammation during HE

**Fig. 4:**
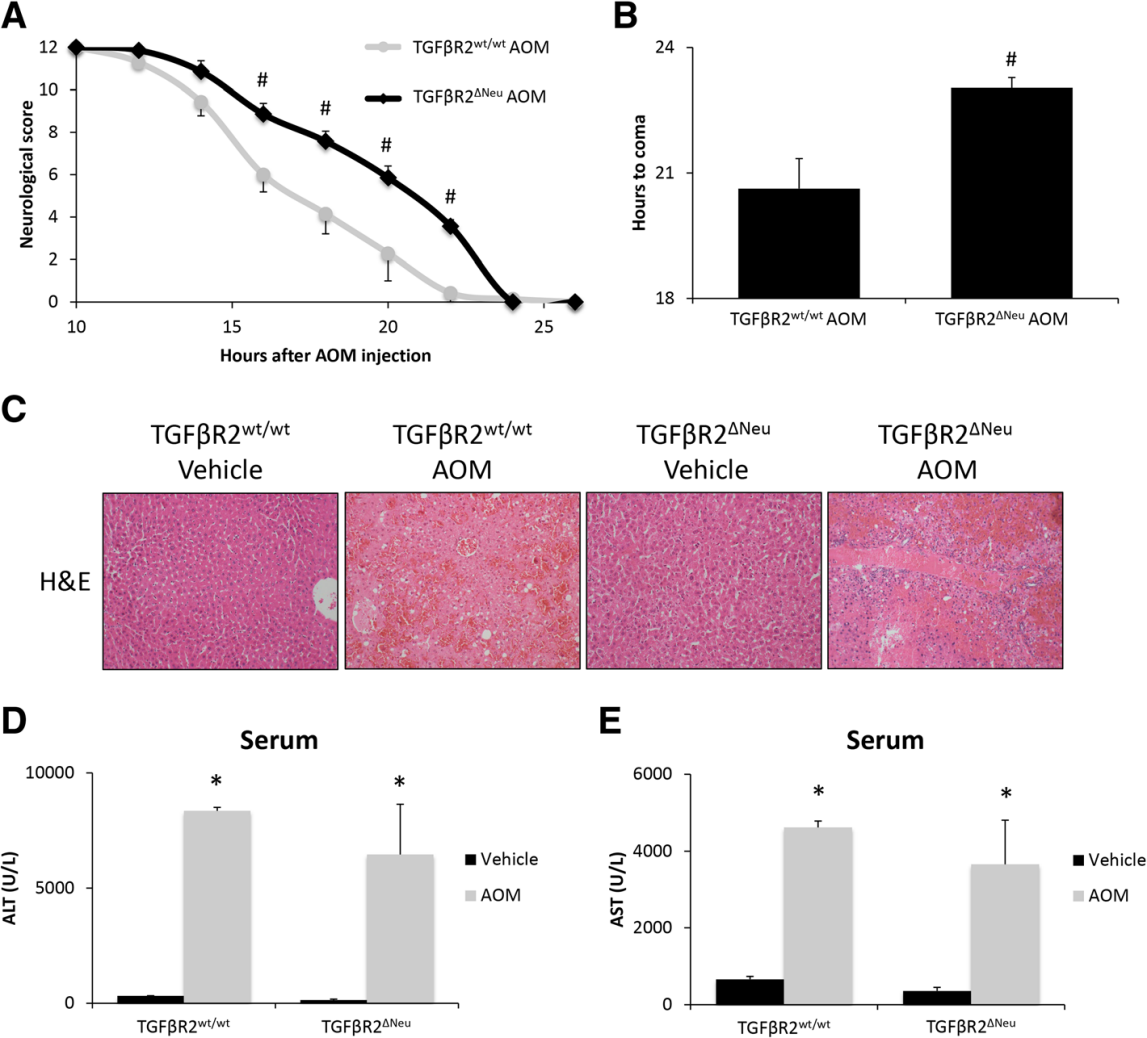
Neuronal TGFβR2 mediates AOM-induced neurological decline. **a** Neurological score analyses as assessed by reflex scores and ataxia at the indicated hours post-AOM injection in TGFβR2^wt/wt^ and TGFβR2^ΔNeu^ mice. **b** Time taken to progress to coma in TGFβR2^wt/wt^ and TGFβR2^ΔNeu^ mice administered AOM. **c** Representative H&E images from the livers of TGFβR2^wt/wt^ and TGFβR2^ΔNeu^ mice administered vehicle or AOM. **d** Serum ALT levels of TGFβR2^wt/wt^ and TGFβR2^ΔNeu^ mice administered vehicle or AOM. **e** Serum AST levels of TGFβR2^wt/wt^ and TGFβR2^ΔNeu^ mice administered vehicle or AOM. *p < 0.05 compared to TGFβR2^wt/wt^ vehicle-treated mice, ^#^*p* < 0.05 compared to TGFβR2^wt/wt^ AOM-treated mice. *n* = 4 for all analyses

We have previously demonstrated that TGFβR2 is expressed in cortical neurons and therefore may be involved with TGFβ1-induced neuroinflammation [12]. AOM-treated TGFβR2^ΔNeu^ mice had a significant reduction in their rate of neurological decline compared to AOM-treated TGFβR2^wt/wt^ mice (*p* < 0.0001) with significant differences between the groups from 16 to 22 h after AOM injection (Fig. 4a). AOM-treated TGFβR2^ΔNeu^ mice had a significantly greater latency in progression to coma compared to AOM-treated TGFβR2^wt/wt^ mice (Fig. 4b). Not surprisingly, there was no change in liver pathology as hepatocyte necrosis, and microvesicular steatosis was present in AOM-treated TGFβR2^wt/wt^ and TGFβR2^ΔNeu^ mice (Fig. 4c). These findings are supported by serum chemistry analyses as ALT (Fig. 4d) and AST (Fig. 4e) are significantly increased in both TGFβR2^wt/wt^ and TGFβR2^ΔNeu^ mice administered AOM with no significant differences between these two groups.

**Fig. 5:**
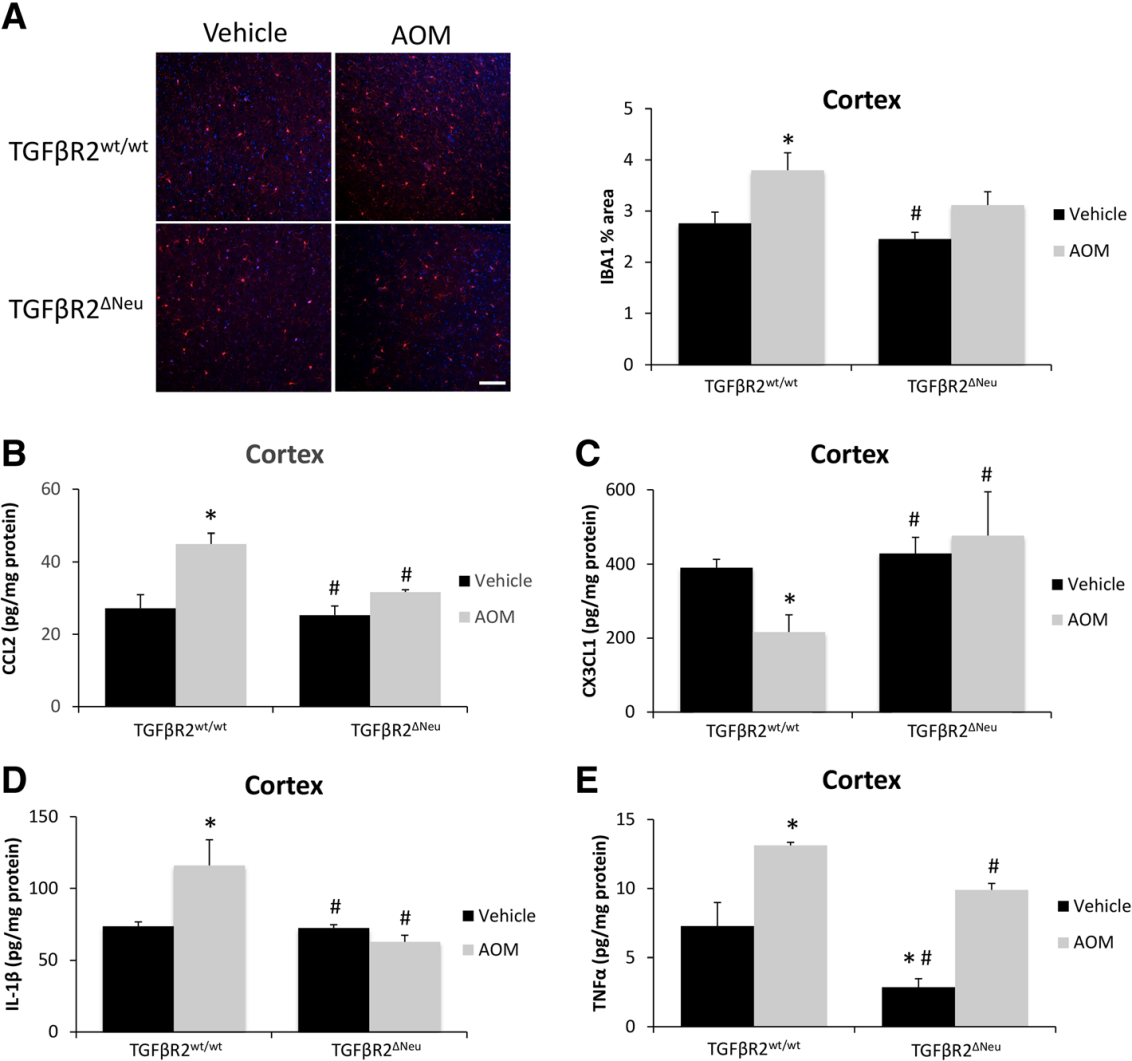
Neuroinflammation during AOM-induced HE is driven by TGFβR2 in neurons. **a** Representative staining and quantification for IBA1 (red) in the cortex from of TGFβR2^wt/wt^ and TGFβR2^ΔNeu^ mice administered vehicle or AOM. DAPI (blue) was used to stain nuclei. Scale bar indicates 75 μM. **b** CCL2 protein expression normalized by total protein concentration in the cortex of TGFβR2^wt/wt^ and TGFβR2^ΔNeu^ mice administered vehicle or AOM. **c** CX3CL1 protein expression normalized by total protein concentration in the cortex of TGFβR2^wt/wt^ and TGFβR2^ΔNeu^ mice administered vehicle or AOM. **d** IL-1β protein expression normalized by total protein concentration in the cortex of TGFβR2^wt/wt^ and TGFβR2^ΔNeu^ mice administered vehicle or AOM. **e** TNFα protein expression normalized by total protein concentration in the cortex of TGFβR2^wt/wt^ and TGFβR2^ΔNeu^ mice administered vehicle or AOM. *p < 0.05 compared to TGFβR2^wt/wt^ vehicle-treated mice, ^#^*p* < 0.05 compared to TGFβR2^wt/wt^ AOM-treated mice. *n* = 3 for all analyses

Genetic deletion of neuronal TGFβR2 could influence neuroinflammation during AOM-induced HE, influencing neurological decline. Microglia proliferation in the cortex was significantly increased in AOM-treated TGFβR2^wt/wt^ mice compared to vehicle-treated TGFβR2^wt/wt^ mice, though this increase was not observed in TGFβR2^ΔNeu^ mice treated with AOM (Fig. 5a). The pro-inflammatory chemokine CCL2 was significantly increased in the cortex of TGFβR2^wt/wt^ AOM-treated mice compared to vehicle-treated TGFβR2^wt/wt^ mice, with TGFβR2^ΔNeu^ AOM-treated mice having no significant change compared to vehicle-treated TGFβR2^wt/wt^ mice (Fig. 5b). The anti-inflammatory chemokine CX3CL1 was significantly decreased in the cortex of TGFβR2^wt/wt^ AOM-treated mice, but not in TGFβR2^ΔNeu^ AOM-treated mice, when both groups were compared to vehicle-treated TGFβR2^wt/wt^ mice, (Fig. 5c). The pro-inflammatory cytokines IL-1β (Fig. 5d) and TNFα (Fig. 5e) were significantly increased in the cortex of TGFβR2^wt/wt^ AOM-treated mice when compared to vehicle-treated TGFβR2^wt/wt^ mice, with TGFβR2^ΔNeu^ AOM-treated mice having no significant increase of either cytokine compared to TGFβR2^wt/wt^ vehicle-treated mice. Therefore, genetic ablation of TGFβR2 in neurons reduced neurological decline and neuroinflammation in AOM-treated mice.

#### Neuroinflammatory response induced by TGFβ1 is dependent upon TGFβ receptor and SMAD3-dependent signaling

**Fig. 6:**
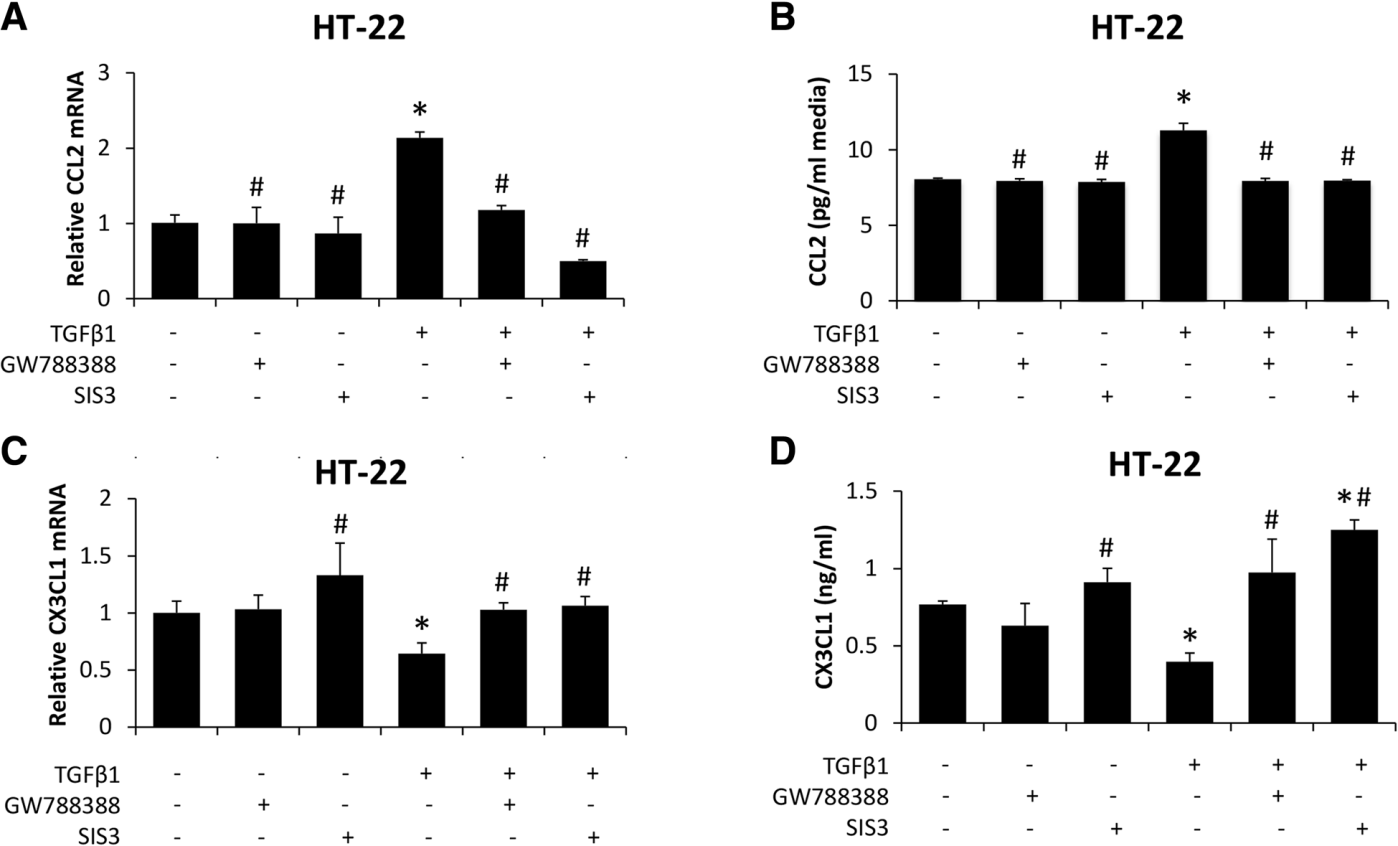
Neuronal CCL2 and CX3CL1 expression is modulated by TGFβ1-mediated signaling. **a** Relative CCL2 mRNA expression of HT-22 cells treated with recombinant TGFβ1, GW788388, or SIS3. **b** CCL2 concentrations in HT-22 cell media treated with recombinant TGFβ1, GW788388, or SIS3. **c** Relative CX3CL1 mRNA expression of HT-22 cells treated with recombinant TGFβ1, GW788388, or SIS3. **d** CX3CL1 concentrations in HT-22 cell media treated with recombinant TGFβ1, GW788388, or SIS3. *p < 0.05 compared to basal HT-22 cells. ^#^*p* < 0.05 compared to TGFβ1-treated HT-22 cells. *n* = 3 for mRNA analyses and *n* = 4 for ELISA analyses

Due to TGFβR2^ΔNeu^ mice being protected from AOM-induced neurological decline and having reduced neuroinflammation compared to TGFβR2^wt/wt^ mice, the mechanisms that lead to the reduced neuroinflammatory response are important to identify. As the activation of microglia can be driven by increased neuronal CCL2 and decreased neuronal CX3CL1 secretion [10, 11, 26, 27], the influence of TGFβ1 on this process was examined in neurons. HT-22 cells were treated with TGFβ1, the TGFβ receptor antagonist GW788388, and the SMAD3 inhibitor SIS3. TGFβ1 treatment alone significantly induced CCL2 mRNA expression (Fig. 6a) and secretion into media (Fig. 6b) compared to basal HT-22 cells, and these effects were significantly inhibited by treatment with GW788388 or SIS3. CX3CL1 mRNA expression (Fig. 6c) and secretion into media (Fig. 6d) were significantly reduced by treatment with TGFβ1 alone in comparison to basal HT-22 cells, and levels were restored to near basal levels if TGFβ1-treated HT-22 cells were co-treated with GW788388 or SIS3.

**Fig. 7:**
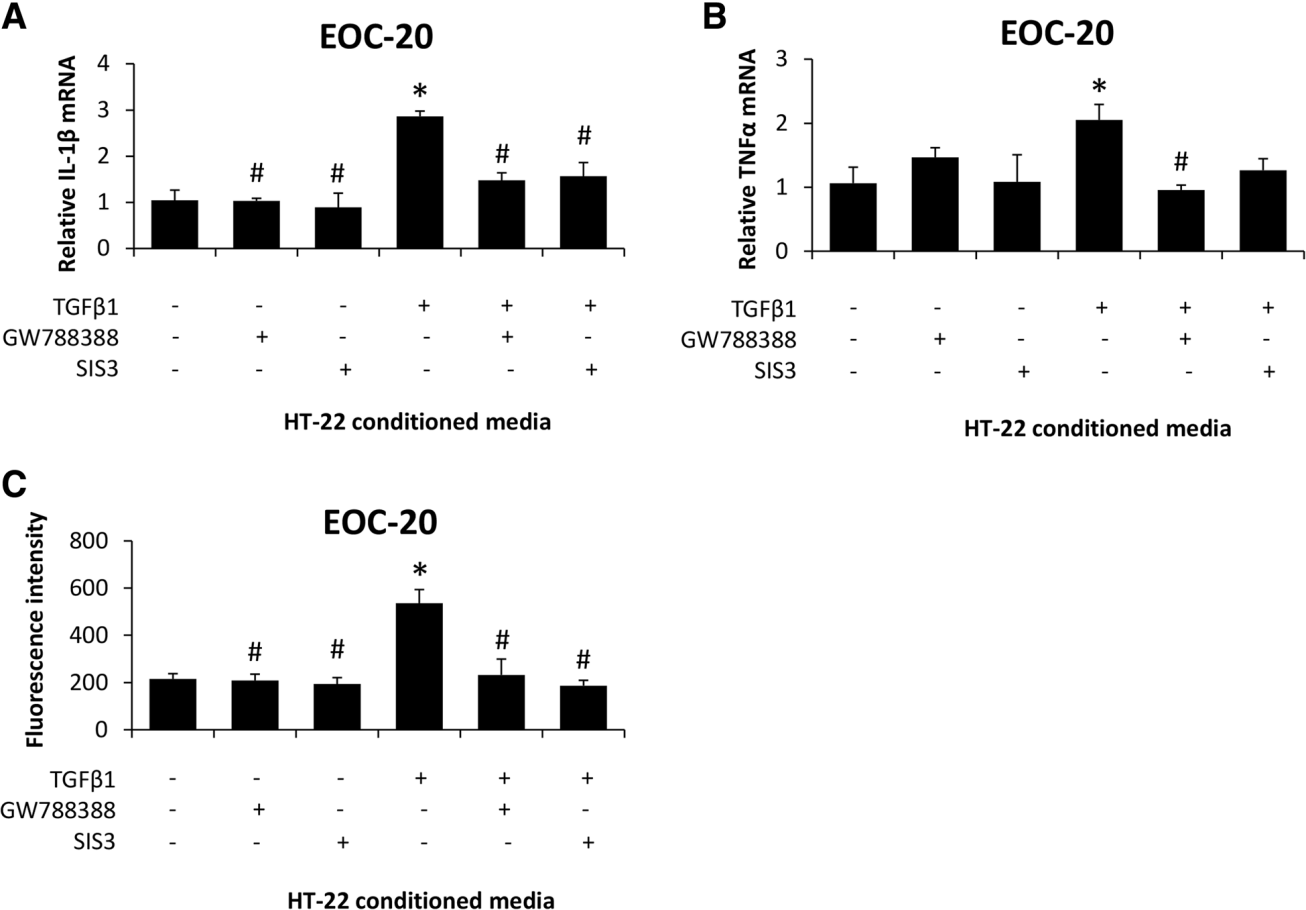
Microglia activation is induced by secreted factors from TGFβ1-stimulated neurons. **a** Relative IL-1β and (**b**) TNFα mRNA expression in EOC-20 cells supplemented with conditioned media from HT-22 cells treated with recombinant TGFβ1, GW788388, or SIS3. **c** Phagocytosis activity, reported as fluorescence intensity of phagocytized *E. coli* bioparticles, in EOC-20 cells supplemented with conditioned media from HT-22 cells treated with recombinant TGFβ1, GW788388, or SIS3. *p < 0.05 compared to EOC-20 supplemented with basal HT-22 conditioned media. ^#^*p* < 0.05 compared to EOC-20 supplemented with TGFβ1-treated HT-22 conditioned media. *n* = 3 for mRNA analyses and *n* = 7 for phagocytosis activity assay

In order to demonstrate that these changes in chemokine expression and secretion would influence microglia activation, conditioned media from HT-22 cells were added to EOC-20 cells, a microglia cell line. Treatment of EOC-20 cells with conditioned media from TGFβ1-treated HT-22 cells increased IL-1β (Fig. 7a) and TNFα mRNA expression (Fig. 7b) when compared to EOC-20 cells supplemented with basal HT-22 conditioned media. In addition, phagocytosis activity (Fig. 7c) was induced in EOC-20 cells supplemented with conditioned media from TGFβ1-treated HT-22 cells compared to EOC-20 cells supplemented with basal-conditioned media from HT-22 cells. These effects of TGFβ1-conditioned media were not observed in conditioned media from HT-22 cells treated with TGFβ1 and GW788388 or TGFβ1 and SIS3 when compared to EOC-20 cells supplemented with basal-conditioned media from HT-22 cells. Together, these data support that TGFβR2 and SMAD3 inhibition in neurons changes the constituents of the conditioned media to inhibit neuroinflammation and phagocytosis.

**Fig. 8:**
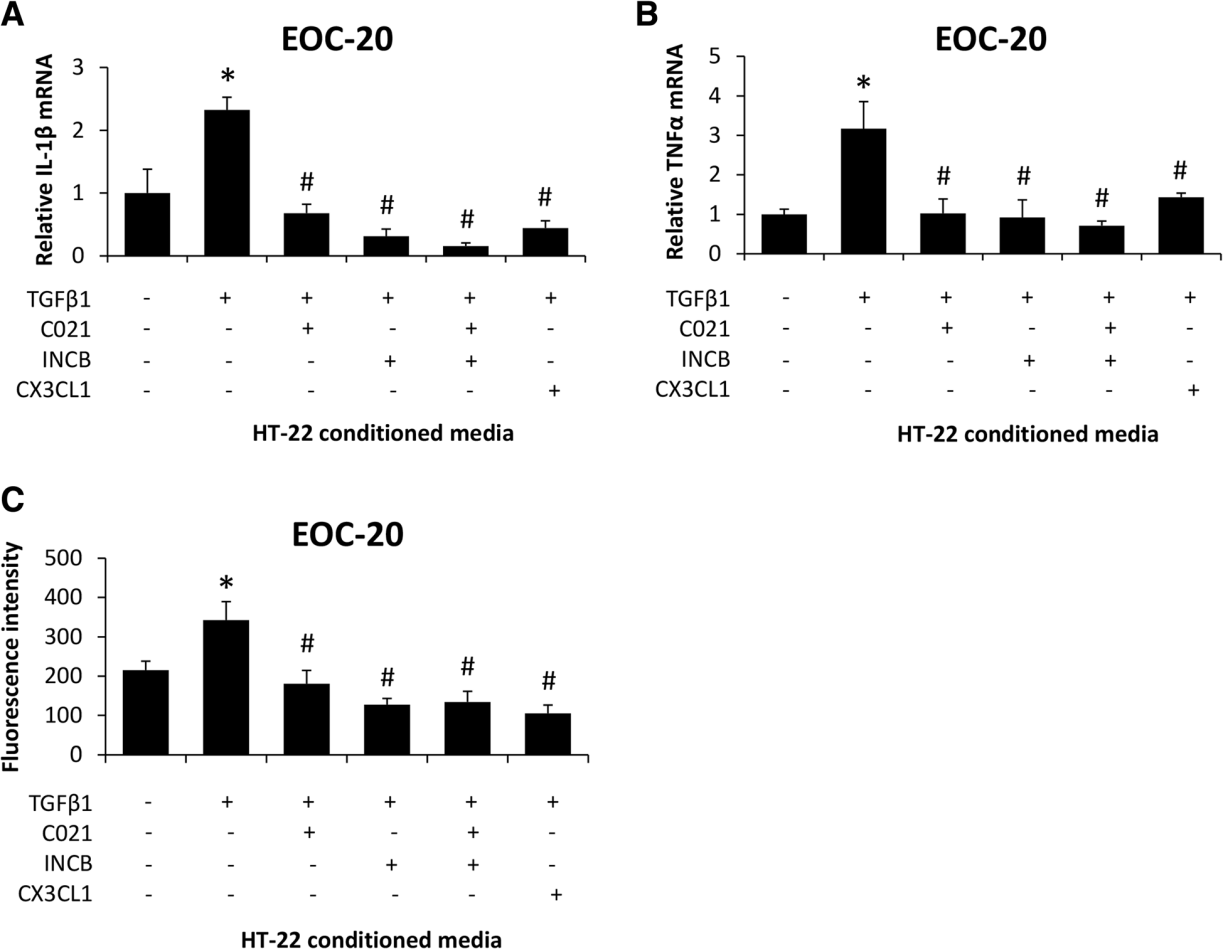
TGFβ1-induced CCL2 and CX3CL1 modulation in neurons promote microglia activation. **a** Relative IL-1β and (**b**) TNFα mRNA expression in EOC-20 cells treated with C021, INCB, or recombinant CX3CL1 following supplementation with conditioned media from HT-22 cells treated with recombinant TGFβ1. **c** Phagocytosis activity, reported as fluorescence intensity of phagocytized *E. coli* bioparticles, in EOC-20 cells treated with C021, INCB, or recombinant CX3CL1 following supplementation with conditioned media from HT-22 cells treated with recombinant TGFβ1. *p < 0.05 compared to EOC-20 supplemented with basal HT-22 conditioned media. ^#^*p* < 0.05 compared to EOC-20 supplemented with TGFβ1-treated HT-22 conditioned media. *n* = 3 for mRNA analyses and *n* = 7 for phagocytosis activity assay

To ensure this was due to changes in CCL2, antagonists to chemokine receptor 2 (INCB) or chemokine receptor 4 (C021) were added to EOC-20 cells supplemented with conditioned media from TGFβ1-treated HT-22 cells. In parallel, CX3CL1 was added into EOC-20 cells supplemented with TGFβ1-treated HT-22 conditioned media. In EOC-20 cells, IL-1β mRNA expression (Fig. 8a), TNFα mRNA expression (Fig. 8b), and phagocytosis (Fig. 8c) were induced by TGFβ1-conditioned media from HT-22 cells, and these effects were not present following the treatment with INCB or C021 as well as following the supplementation of CX3CL1 when all groups were compared to EOC-20 cells supplemented with basal-conditioned media from HT-22 cells. Together, these data support that TGFβ1 increases CCL2 and decreases CX3CL1 secretion in neurons, which directly stimulates neuroinflammation and phagocytosis activity in microglia.

## Discussion

**Fig. 9:**
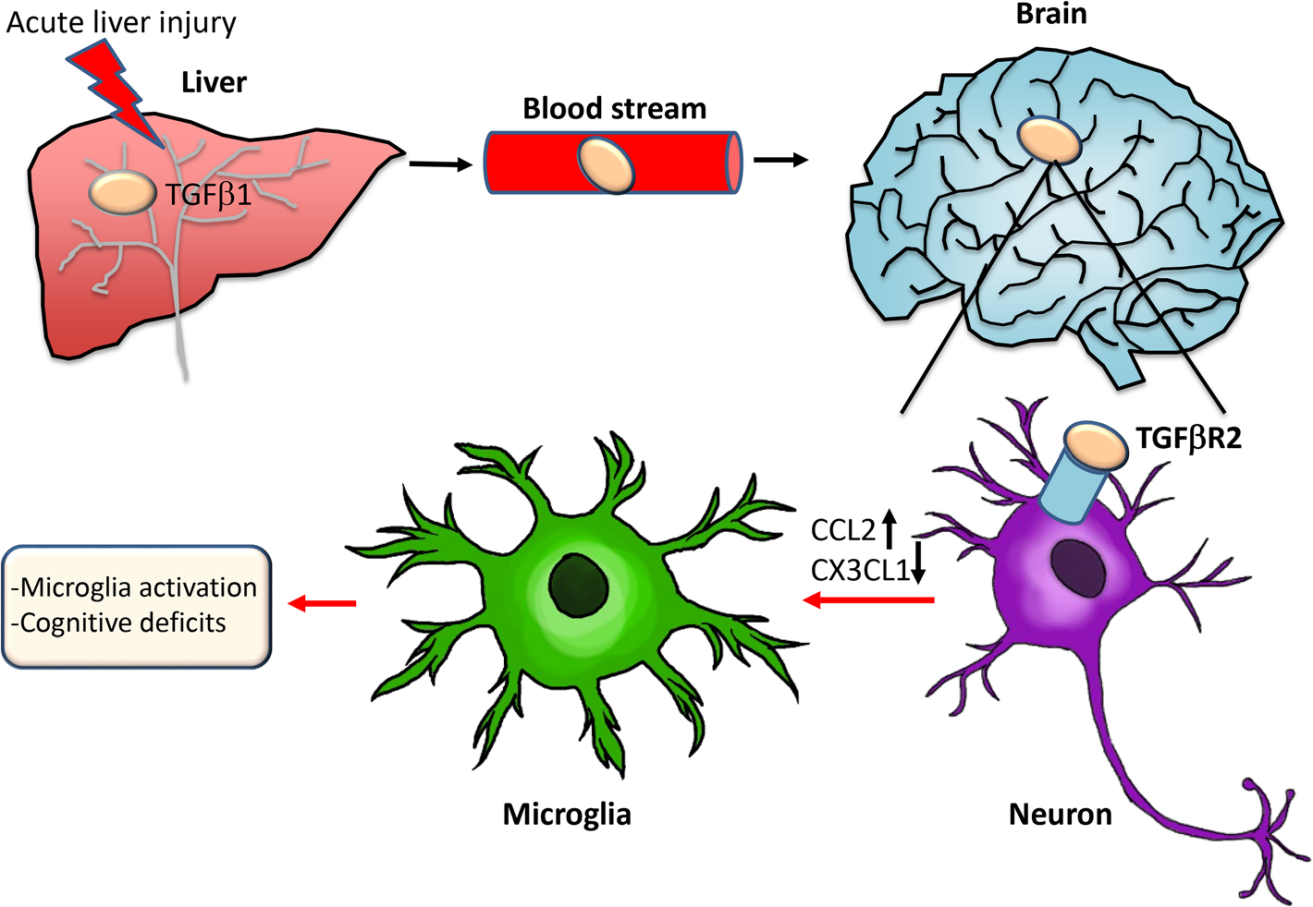
Working model of TGFβ1 signaling during AOM-induced HE. AOM-induced liver failure leads to an increase of hepatic TGFβ1 that enters the bloodstream, crosses the blood–brain barrier, and enters the brain. This results in the activation of TGFβR2 on neurons leading to increased expression and secretion of CCL2 and decreased expression and secretion of CX3CL1 from neurons. This imbalance of CCL2 and CX3CL1 leads to changes in chemokine receptor-mediated signaling in microglia, which ultimately results in microglia activation and worse HE outcomes

The data presented here indicates that TGFβ1 plays a role in the pathogenesis of HE by inducing microglia activation and neuroinflammation. TGFβ1 is released into the circulation after acute liver failure and binds TGFβR2 in neurons, resulting in increased CCL2 expression and decreased CX3CL1 expression leading to microglial activation. A summary of this disease process is provided as a working model (Fig. 9). Strategies that decrease circulating TGFβ1 or antagonize neuronal TGFβR2 may be effective therapeutic options for the management of HE.

We have previously demonstrated that hepatic TGFβ1 expression is upregulated in the AOM model of acute liver failure [12], which is consistent with other studies in response to a variety of hepatotoxins [28, 29]. During acute liver failure in patients due to acetaminophen toxicity, increased levels of hepatic TGFβ1 mRNA expression and circulating TGFβ1 levels have been observed when compared to healthy controls [30]. In the current study, we observed increased TGFβ1 expression localized primarily to hepatocyte populations due to its co-localization with CK8, supporting findings from our previous work demonstrating TGFβ1 co-localization> with albumin during AOM-induced liver failure [12]. Possible consequences of TGFβ1 signaling in hepatocytes may range from induction of apoptotic pathways [31] to the inhibition of hepatocyte proliferation and liver regeneration [29]. In the current study, we have demonstrated that anti-TGFβ1 treatment had similar liver pathology after injection of AOM compared to IgG1 control mice, but had protective effects on the neurological complications associated with acute liver failure. These data suggest that while hepatic TGFβ1 expression and circulating TGFβ1 levels are increased in response to AOM-induced liver injury, this cytokine has minimal involvement in the subsequent hepatocyte apoptotic/regeneration pathways in this model. These observations are not surprising given that AOM-induced liver injury is predominantly due to hepatocyte necrosis, rather than apoptosis, with minimal regeneration occurring [5, 32], offering a unique model of liver injury allowing for the dissection of hepatocyte versus neurological actions of TGFβ1.

The data presented here suggest that increased circulating TGFβ1 following AOM-induced hepatic necrosis is influencing neurological function and neuroinflammation during acute liver failure. The concept that systemic inflammatory signals can induce neuroinflammation is not without precedent in that microglia activation and astrogliosis have been observed in many models of systemic inflammation [33–36], although the mechanism by which this occurs is uncertain. In the AOM model, we observe increased circulating TGFβ1 expression following liver injury. In previous studies, we found increased TGFβ1 protein expression in the cortex without a subsequent increase of mRNA expression [12], which supports that systemic increases of TGFβ1 lead to elevated TGFβ1 levels in the brain. While we do observe increased levels of TGFβ1 in the blood, it is not known whether this is free TGFβ1, TGFβ1 bound to plasma proteins, or TGFβ1 present in extracellular vesicles. One possible mechanism for the induction of neuroinflammation by TGFβ1 is through circulating exosomes [34]. Indeed, exosomes isolated from the bloodstream in a mouse model of endotoxemia contained many inflammatory microRNAs and cytokines that induced systemic inflammation and neuroinflammation when injected into a naïve mouse [34]. Given that TGFβ1 can be packaged into exosomes [37, 38], it is conceivable that the systemic TGFβ1 observed in our model of acute liver failure may be delivered via exosomes. Regardless of the mechanism of delivery, for circulating TGFβ1 to influence neuroinflammation, TGFβ1 has to cross the blood–brain barrier. During the pathogenesis of HE, the blood–brain barrier becomes hyper-permeable to many molecules [39, 40]. Indeed, we have demonstrated that TGFβ1 itself is able to induce hyperpermeability of the blood–brain barrier during acute liver failure via the induction of matrix metalloproteinase-9 expression in brain endothelial cells and the alteration of tight junction protein expression [13]. Therefore, the strong effects observed as a result of increased TGFβ1 signaling regarding neuroinflammation during AOM-induced HE may partially result from the entry of other signaling mediators and metabolites, like bile acids, into the brain.

TGFβ1 signaling in the brain, similar to other organs, can be pro- or anti-inflammatory depending upon the disease state [41–44]. For example, TGFβ1 inhibits microglia chemotaxis towards amyloid beta aggregates observed in Alzheimer’s disease via activation of TGFβR/SMAD-2-mediated downregulation of the chemokine CCL5 [41]. Conversely, TGFβ1 signaling via TGFβR/SMAD-2/3 activation is pro-inflammatory in the context of mild traumatic brain injury [44]. In vitro studies using primary mouse and rat microglia treated with rTGFβ1 find differential effects on physiology and gene expression [45]. The proliferation of microglia was stimulated by TGFβ1 treatment in rat microglia but not mouse microglia [45]. In addition, cytokine profiles were changed in mouse microglia in response to TGFβ1 but were not consistent with the anti-inflammatory cytokine IL-10 being upregulated while the anti-inflammatory peroxisome proliferator-activated receptor gamma was downregulated [45]. The data presented in the current study support a role for TGFβ1 in microglia activation and the subsequent neuroinflammatory processes. That being said, these findings may not translate into chronic models of HE and further studies are necessary to fully characterize the role of TGFβ1 signaling with neuroinflammation during HE.

We have previously demonstrated that CCL2 expression and secretion from neurons is central to the activation of microglia and subsequent pro-inflammatory cascade in the brain during acute liver failure [10]. Furthermore, we have previously demonstrated that TGFβR2 expression is found predominantly in neurons in the brain and that TGFβ signaling increases the activation of SMAD3 in neurons during acute liver failure [12]. In the current study, we present novel data indicating that neuron-specific knockout of TGFβR2 attenuated the expression of CCL2, increased CX3CL1 expression, inhibited microglia activation, and reduced neurological deficits during acute liver failure. These observations are consistent with previous studies using models of inflammation in the liver and other organs [11, 15, 46–48]. In vitro studies determined that TGFβ1 led to increased CCL2 and decreased CX3CL1 secretion for HT-22 cells, and this resulted in a direct increase of a neuroinflammatory response for EOC-20 cells, supporting the in vivo findings of this study. That being said, the role of astrocytes in neuroinflammation during AOM-induced HE was not investigated, and these cells could play a contributing role in this process. In human astrocytes stimulated with IL-1β and TNFα, the same cytokines we show upregulated in the cortex of our AOM-treated mice; 14 chemokines/cytokines were upregulated while 2 were downregulated [49]. Therefore, even though our manipulations were primarily targeted to TGFβR2 activity on neurons, it is possible that astrocytes contribute to these results.

## Conclusions

In summary, the data presented here suggest that during acute liver failure, TGFβ1 expression is increased in the liver, contributing to increased circulating TGFβ1, which in turn regulates the neuroinflammation and neurological deficits associated with HE. Strategies to neutralize TGFβ1 in the bloodstream and inhibit neuronal TGFβR2 activity are able to attenuate the development of HE without significantly altering the underlying liver damage in this model of HE. Together, these data suggest that TGFβ1 may be a central pro-inflammatory mediator contributing to neurological impairment during HE, and strategies to target TGFβ1 signaling may be a viable target for the development of novel adjunct therapies for the management of HE.

## Additional file

Additional file 1: Supplementary Materials and Methods. **Table S1.** Primers used for PCR. Supplementary Results. **Figure S1.** Validation of TGFβR2^wt/wt^ as appropriate HE controls. **Figure S2.** Immunofluorescence images for DAPI and YFP in TGFβR2^wt/wt^ and TGFβR2^ΔNeu^ cortex. (DOCX 3171 kb)

## Abbreviations

ALT: Alanine aminotransferase
Anti-TGFβ1: Neutralizing antibodies against TGFβ1
AOM: Azoxymethane
AST: Aspartate aminotransferase
C021: C 021 dihydrochloride
CCL2: Chemokine ligand 2
CX3CL1: C-X3-C motif ligand 1
DAPI: 4′,6-diamidino-2-phenylindole
GAPDH: Glyceraldehyde 3-phosphate dehydrogenase
HE: Hepatic encephalopathy
IgG1: Immunoglobulin G1
IL-1ß: Interleukin-1 beta
INCB: INCB 3284 dimesylate
TGFβ: Transforming growth factor beta
TGFβR2: Transforming growth factor beta receptor 2
TNFα: Tumor necrosis factor alpha
