# Supplementary material for: Elevated circulating TGFβ1 during acute liver failure activates TGFβR2 on cortical neurons and exacerbates neuroinflammation and hepatic encephalopathy in mice
Source: J Neuroinflammation. 2019 Apr 2;16:69. doi: 10.1186/s12974-019-1455-y (PMC6446280; doi:10.1186/s12974-019-1455-y)
Supplement: Supplementary file 1 — Supplementary Materials and Methods. Table S1. Primers used for PCR. Supplementary Results. Figure S1. Validation of TGFβR2wt/wt as appropriate HE controls. Figure S2. Immunofluorescence images for DAPI and YFP in TGFβR2wt/wt and TGFβR2ΔNeu cortex. (DOCX 3171 kb) [file 12974_2019_1455_MOESM1_ESM.docx]

**Additional file 1**

**Elevated circulating TGFβ1 during acute liver failure activates TGFβR2 on cortical neurons and exacerbates neuroinflammation and hepatic encephalopathy in mice**

Matthew McMillin^1,2, 4^, Stephanie Grant^1,2,3^, Gabriel Frampton^1,2,4^, Anca D. Petrescu^1,2,3^, Elaina Williams^1,2^, Brandi Jefferson^1,2^, Alison Thomas^2^, Ankita Brahmaroutu^2^, Sharon DeMorrow^1,2, 3, 4*^

^1^Central Texas Veterans Health Care System, Temple, TX, United States, ^2^Texas A&M University Health Science Center, College of Medicine, Department of Medical Physiology, Temple, TX, United States, ^3^Division of Pharmacology and Toxicology, College of Pharmacy, University of Texas Austin, and ^4^Department of Internal Medicine, Dell Medical School, University of Texas Austin.

**Table of Contents**

## Supplementary Materials and Methods…………………………………………..…3

Supplementary Table 1……………………………………………………….………4

## Supplementary Results………………………………………………...……………..4

## Supplementary Fig. 1………………………………………………...……………….6

## Supplementary Fig. 2………………………………………………...……………….7

**Supplementary Materials and Methods**

GENOTYPING

Tail snips were taken from mice and DNA was isolated using a DNeasy Blood & Tissue Kit (Qiagen, Germantown, MD) according to the manufacturer’s instructions. DNA was quantified using a Nanodrop 2000 spectrophotometer (Thermo Fisher Scientific, Waltham, MA). PCR was performed using the primers in Table 1 using a 3-step amplification using 94°C, 60°C and 72°C steps for 30 cycles. Samples were mixed with 5 µL ethidium bromide and loaded on a 2% agarose gel. Gels were imaged using a LiCor Odyssey FC imaging system (Lincoln, NE).

IMMUNOFLUORESCENCE AND IMAGING YFP REPORTER

Brain sections were cut into 30 μm brain sections. Brain sections were placed into 24-well plates and were subsequently washed with 0.1% phosphate buffered saline containing Triton X-100 (MilliporeSigma, Burlington, MA). Brain sections were blocked with 5% goat serum at room temperature for 2 hours and then incubated in a TGFβR2 antibody (MilliporeSigma) overnight. Cells were washed with phosphate buffered saline and incubated with anti-rabbit Cy3 (Jackson ImmunoResearch (West Grove, PA) for 2 hours at room temperature. After a second phosphate buffered saline wash, brains were moved to positively charged slides and had coverslips mounted using ProLong© Gold Antifade Reagent containing 4',6-diamidino-2-phenylindole. Brain sections were viewed and imaged using a Leica TCS SP5-X inverted confocal microscope (Leica Microsystems, Buffalo Grove, IL).

**Table S1:** *Primers used for PCR*

| **Gene Name** | **Forward Primer (5’-3’)** | **Reverse Primer (5’-3’)** |
| --- | --- | --- |
| ***Floxed TGFβR2*** | **TAT GGA CTG GCT GCT TTT GTA TTC** | **TGG GGA TAG AGG TAG AAA GAC ATA** |
| ***Thy1-Cre*** | **TCT GAG TGG CAA AGG ACC TTA GG CAA** | **CGC TGA ACT TGT GGC CGT TTA CG GTC** |
| **Thy1-Cre**  **Transgene Control** | **CAA ATG TTG CTT GTC TGG TG** | **GTC AGT CGA GTG CAC AGT TT** |

**Supplementary Results**

VALIDATION OF MOUSE MODELS

All mice used for tissue collection or behavioral analyses had their genotype validated using PCR. In order to ensure that TGFβR2^wt/wt^ were appropriate controls for this study, AOM experiments were performed using TGFβR2^fl/fl^ mice and TGFβR2^wt/wt^ mice treated with tamoxifen and compared them to TGFβR2^ΔNeu^ treated with corn oil and TGFβR2^ΔNeu^ treated with tamoxifen. We observed no changes in neurological decline (Supplemental Fig 1A) or time to coma (Supplemental Fig 1B) with there being no significant differences between TGFβR2^fl/fl^ mice and TGFβR2^wt/wt^ mice treated with tamoxifen and TGFβR2^ΔNeu^ treated with corn oil. In addition, all three of these groups had significantly worse neurological decline and time to coma compared to TGFβR2^ΔNeu^ treated with tamoxifen. Therefore, in the context of these experiments, HE progression is similar in C57Bl/6 mice compared to transgenic Thy1-cre mice.

Thy1-Cre mice have a modified Thy1 promotor containing both a CreER^T2^ fusion protein and enhanced yellow fluorescent protein (YFP), which allows them to express Cre recombinase and YFP when injected with tamoxifen. YFP expression was found to be present only in mice treated with tamoxifen with no apparent differences in vehicle or AOM-treated mice, validating Thy1-Cre expression in these mice (Supplemental Fig 2A). In order to assess that Thy1-Cre expression led to a functional decrease in TGFβR2 expression, immunofluorescence for TGFβR2 was performed in the cortex of TGFβR2^wt/wt^ and TGFβR2^ΔNeu^ mice injected with tamoxifen (Supplemental Fig 2B), demonstrating that neuronal TGFβR2 was knocked down only in the TGFβR2^ΔNeu^ mice in response to tamoxifen.

**
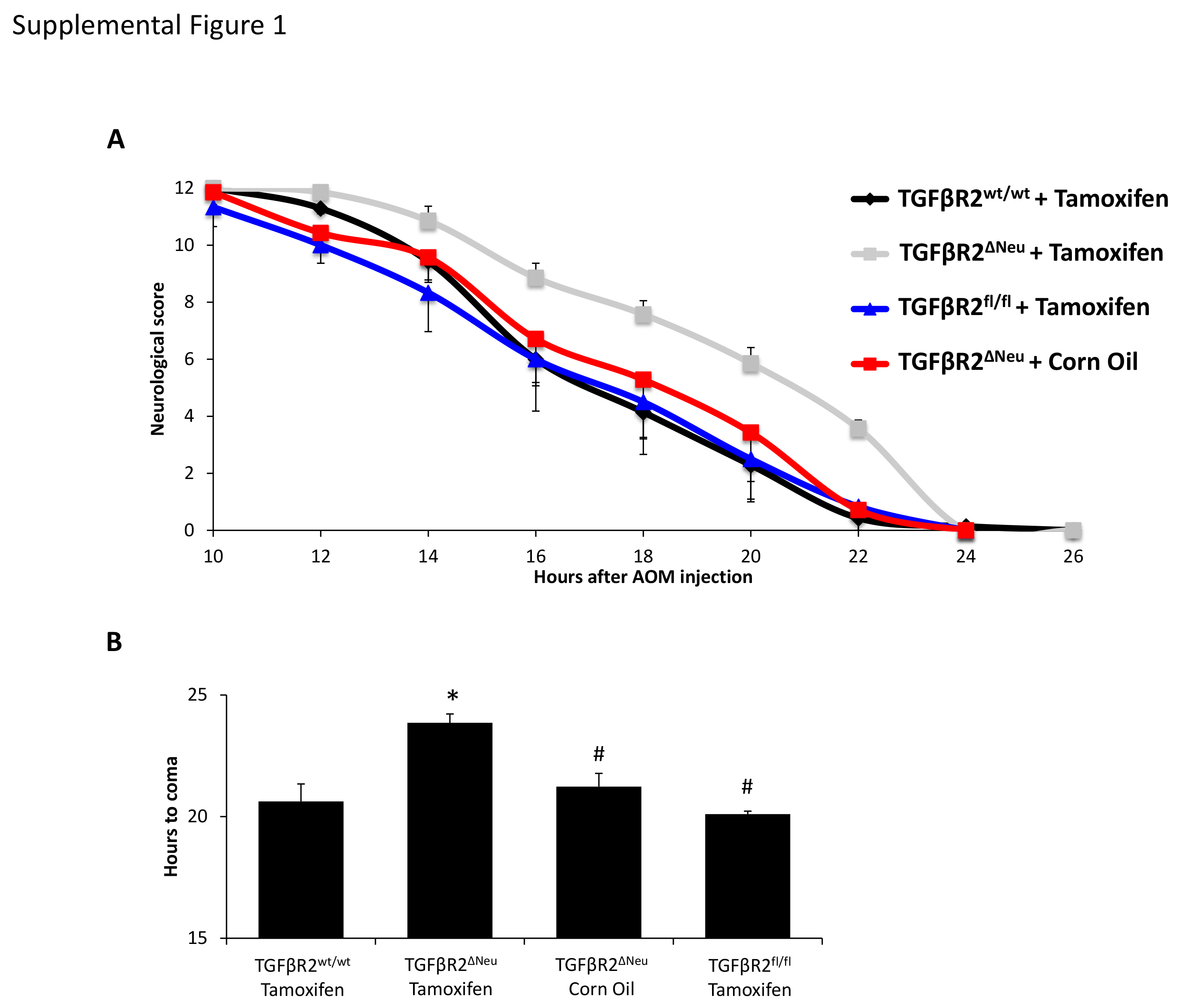
**

**Figure S1: Validation of TGFβR2^wt/wt^ as appropriate HE controls.** (A) Neurological decline in TGFβR2^fl/fl^ mice, TGFβR2^wt/wt^ mice and TGFβR2^ΔNeu^ treated with tamoxifen and TGFβR2^ΔNeu^ treated with corn oil. (B) Time to coma expressed in hours in TGFβR2^fl/fl^ mice, TGFβR2^wt/wt^ mice and TGFβR2^ΔNeu^ treated with tamoxifen and TGFβR2^ΔNeu^ treated with corn oil. *=p<0.05 compared to TGFβR2^wt/wt^ treated with tamoxifen. #=p<0.05 compared to TGFβR2^ΔNeu^ treated with tamoxifen. n=4 per group.

**
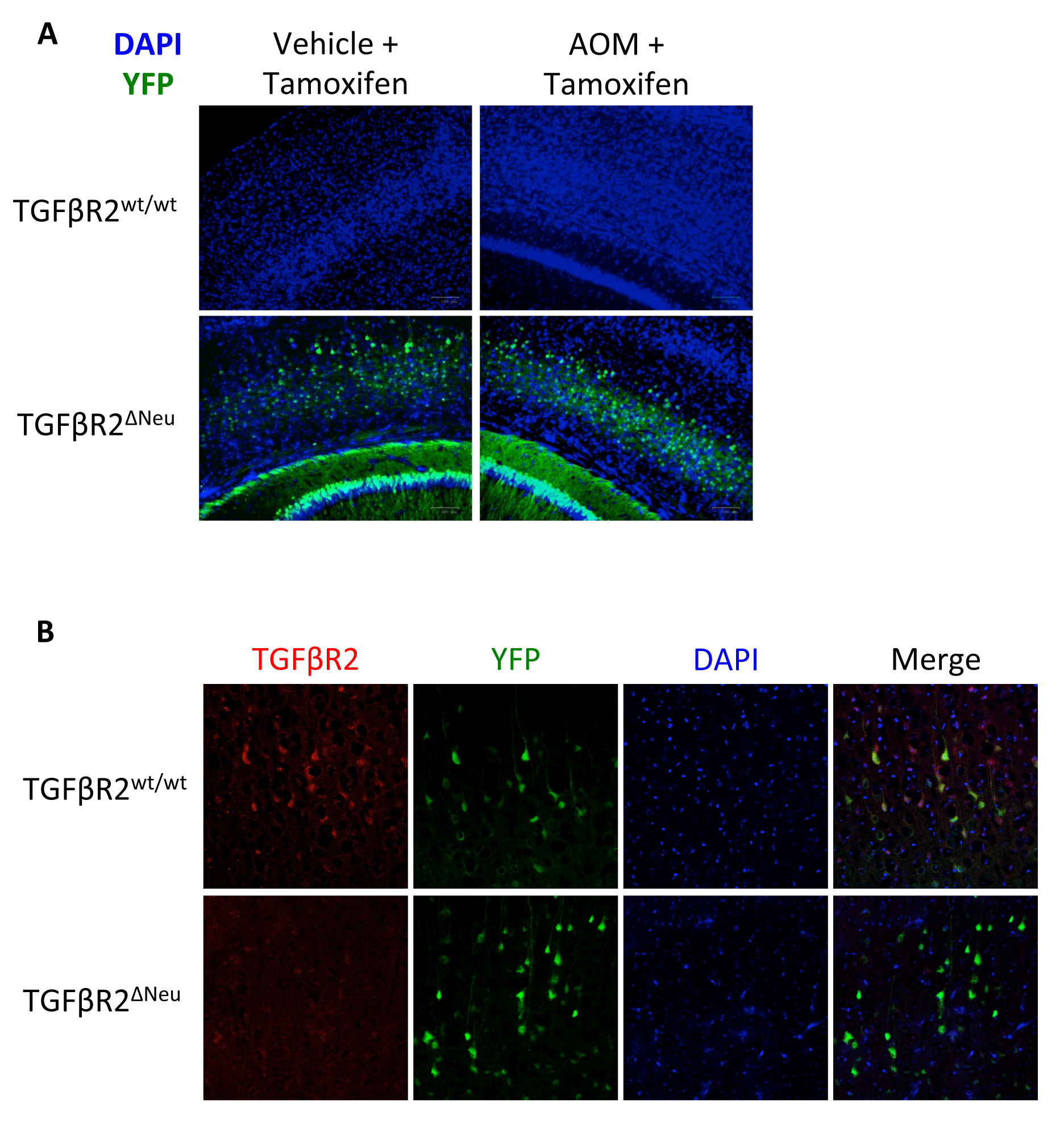
**

**Figure S2: Immunofluorescence images for DAPI and YFP in TGFβR2^wt/wt^ and TGFβR2^ΔNeu^ cortex.** (A) TGFβR2^wt/wt^ and TGFβR2^ΔNeu^ mice were injected with tamoxifen prior to injection of vehicle or AOM. Green staining is for yellow fluorescent protein and blue staining is for DAPI. (B) TGFβR2^wt/wt^ and TGFβR2^ΔNeu^ mice were injected with tamoxifen and stained for TGFβR2 (red). Yellow fluorescent protein is shown in green and DAPI is shown in blue.
